# Chronic intermittent hypoxia‐mediated cognitive dysfunction in ovariectomized rats

**DOI:** 10.1113/EP092018

**Published:** 2025-05-19

**Authors:** Emily C. Cheung, Joan B. Escobar, Bridget R. Alber, Caitlin Ribeiro, Ishan Abdullah, Grant Kowalik, Jeannette Rodriguez, Grey Harral, Makeda Melkie, Aman Gill, John T. Ketzenberger, John Bethea, Vsevolod Y Polotsky, Vivek Jain, Kathryn Schunke, Matthew W. Kay, David Mendelowitz

**Affiliations:** ^1^ Department of Pharmacology and Physiology George Washington University Washington District of Columbia USA; ^2^ Department of Biomedical Engineering George Washington University Washington District of Columbia USA; ^3^ Department of Anatomy and Cell Biology George Washington University Washington District of Columbia USA; ^4^ Department of Anesthesiology and Critical Care Medicine George Washington University Washington District of Columbia USA; ^5^ Department of Medicine George Washington University Washington District of Columbia USA; ^6^ Department of Cell & Molecular Biology University of Hawaii Honolulu Hawaiʻi USA

**Keywords:** Alzheimer's disease, Morris water maze, obstructive sleep apnoea, ovariectomy, plethysmography, respiration

## Abstract

Obstructive sleep apnoea (OSA) is a prevalent cardiorespiratory disorder associated with significant neurocognitive consequences. Despite the higher prevalence of OSA in men, there is a strong association between OSA and Alzheimer's disease (AD), which disproportionately affects women. This study aimed to investigate the impact of chronic intermittent hypoxia (CIH), a hallmark of OSA, on cognitive function and AD markers in ovariectomized, female rats. At 8 weeks of age, 16 Sprague–Dawley rats underwent ovariectomy and were exposed to CIH for 26 weeks. Cognitive function was assessed using the Morris water maze, revealing significant deficits in spatial learning (*P *< 0.0001) and memory (*P* = 0.008) in CIH‐exposed rats, compared to controls. Analysis of hippocampal tissue showed increased total tau protein (*P* = 0.0078), indicative of AD pathology. Additionally, CIH‐exposed rats exhibited respiratory dysfunction characterized by increased frequency of apnoeas (*P* = 0.0328). These findings provide preclinical evidence of the association between OSA, cognitive decline and AD pathology in females, emphasizing the importance of sex‐specific research in understanding and addressing these pathophysiological interconnections.

## Abstract (highlights)

- **What is the central question of this study?**
  Does the cumulative effects of long‐term exposure to chronic intermittent hypoxia (CIH), high‐fat diet and ovariectomy in female rats lead to cognitive decline and respiratory dysfunction?
- **What is the main finding and its importance?**
  Long‐term exposure to CIH caused learning and memory impairments, which were correlated to increased tau protein. Respiratory analysis revealed an increase in apnoeic events in CIH animals.

## 1 INTRODUCTION

Obstructive sleep apnoea (OSA) is a highly prevalent, yet underdiagnosed cardiorespiratory disorder, affecting 25–30% of men and 9–17% of women in the United States (Slowik et al., 2024). Characterized by recurrent episodes of obstruction in the upper airway during sleep, OSA impacts sleep architecture and tissue oxygenation, which can give rise to a cascade of consequences spanning cardiovascular, metabolic and neurocognitive domains.

The relationship between OSA and Alzheimer's disease (AD) is bi‐directional. Hypoxia exposure promotes neurodegenerative changes and dementia, and those negatively impact respiratory regulation and drive (Bubu et al., 2017; Daulatzai, 2015; Emamian et al., 2016; Wrzesień et al., 2024; Zhang et al., 2013). Three of the defining characteristics of OSA, intermittent hypoxia, hypertension and sleep fragmentation, have also been independently identified as high‐risk factors for the occurrence of AD (Bubu et al., 2017; Daulatzai, 2015; Emamian et al., 2016; Zhang et al., 2013). OSA nearly doubles the risk for cognitive decline and/or AD compared to age‐matched individuals without OSA (Jean‐Louis et al., 2008). OSA is primarily treated with continuous positive airway pressure (CPAP), and CPAP withdrawal for just three nights increased circulating AD biomarkers (Kam et al., 2022). In addition to cognitive decline, AD can impact brain morphology, as it is commonly characterized by the aggregation of extracellular amyloid‐beta plaques and intracellular hyperphosphorylation of tau protein in the hippocampal region (Busche & Hyman, 2020).

Chronic intermittent hypoxia (CIH), a key clinical feature of OSA, has been widely used in preclinical models (Cheung et al., 2024; Jameson et al., 2016; Rodriguez et al., 2023). Animals exposed to long‐term (at least a month) daily CIH present with similar cardiovascular and neurological deficits as those described for OSA. CIH has also been shown to increase tau hyperphosphorylation in the hippocampus in rats (Marciante et al., 2022) and impair learning and memory in male mice (Li et al., 2022). Similarly, CIH increased AD‐related pathogenic molecular signalling in male P301S human mutant tau mice (Kazim et al., 2022). In humans, CIH increased pathological tau seeding, phosphorylated tau load, and deficits in memory and synaptic plasticity (Bubu et al., 2019).

Most preclinical studies of associations between CIH exposure, the presence of AD markers, and the occurrence of cognitive decline have been conducted in male rats (Ng et al., 2010; Snyder et al., 2018, 2017; Yang et al., 2012; Yuan et al., 2015) and male mice (Kazim et al., 2022; Shiota et al., 2013). There are considerably fewer studies in females. Understanding these associations in female preclinical models is critical, given that women are disproportionately affected by AD or related dementia, which has been linked to age‐related neurohormonal changes at the onset of or after menopause (Mosconi et al., 2018). In this study, we investigated whether clinically relevant risk factors, modelled by ovariectomy and CIH, generate symptoms and markers of AD. We measured respiratory parameters, cognitive function using the Morris water maze (MWM), and histopathological markers of AD, including amyloid beta plaques and tau accumulation.

## 2 METHODS

### 2.1 Ethical approval

All animal procedures were approved by the George Washington University Institutional Animal Care and Use Committee and compliant with the Panel of Euthanasia of the American Veterinary Medical Association and the National Institutes of Health (NIH) *Guide for the Care and Use of Laboratory Animals* (GWU IACUC no. 2022‐028). All efforts were made to minimize animal pain and suffering.

### 2.2 Animal preparation

**FIGURE 1:**
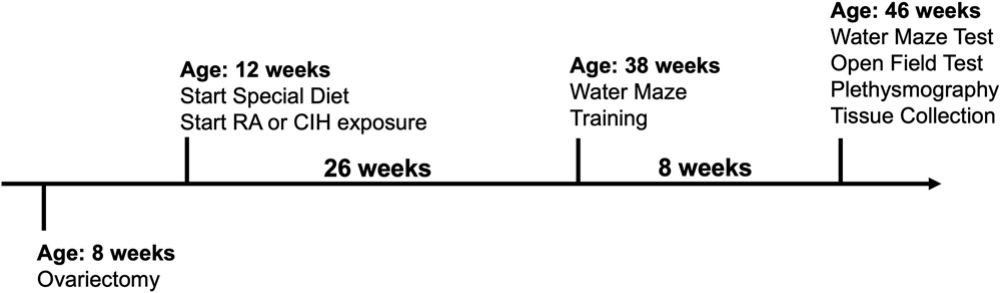
Animal preparation and timeline. At 8 weeks of age, female SD rats were ovariectomized. At 12 weeks, they switched diets from normal chow to high‐fat diet (26% fructose, 1.5% NaCl adj calories diet) and began daily exposures to either RA or CIH (cycled between room air (21% O_2_, 79% N_2_) and hypoxia (6% O_2_, 94% N_2_), for 8 h/day for 26 weeks). At 38 weeks, the animals underwent water maze training. Eight weeks later, they underwent a series of tests, including the water maze test, open field test and whole‐body plethysmography, prior to sacrifice and tissue collection. CIH, chronic intermittent hypoxia; RA, room air.

Female Sprague–Dawley rats (Hilltop Lab Animals, Inc., Scottdale, PA, USA) were ovariectomized at 8 weeks of age (250–300 g) and housed in pairs thereafter (*n* = 16) (Figure 1). For the ovariectomy, animals were anaesthetized with isoflurane (5% for induction, 2% for maintenance). The depth of anaesthesia was monitored during the surgery by assessing the pedal reflex. Pre‐ and post‐surgical analgesia (subcutaneous buprenorphine HCl (Covetrus, Portland, ME, USA) injection, 0.01 mg/kg every 12 h for 3 days) was administered. During the procedure, the animal was placed in the supine position and a 1.5 cm incision was made on the abdominal wall. Adipose tissue was temporarily lifted to expose the ovaries. For each ovary, the distal uterine horn and ovarian blood vessels were ligated (Park et al., 2010). All connections to the ovary were severed, and the ovary was removed. Proper aseptic techniques were maintained throughout this procedure. After surgery, animals were housed in pairs in the institutional animal vivarium under a 12:12 light–dark cycle with unrestricted access to food and water. Four weeks after ovariectomy, the diet was switched from standard chow to a high‐fat diet consisting of 55% fat, 8.5% sucrose and 1.5% NaCl (TD210254, Envigo Teklad, Madison, WI, USA) to induce obesity.

### 2.3 Experimental groups

Four weeks after ovariectomy, each cage was randomly assigned to either a normoxic (room air non‐CIH, *n* = 8) or hypoxic group (CIH, *n* = 8). Animals were transported from the vivarium to another room to spend 8 h (between 11.00 h and 19.00 h, aligning with the rodent sleep period) under exposure to room air or CIH. CIH animals, inside their cages with normal bedding and unrestricted access to food and water, were placed inside sealed commercial chambers with computer‐controlled atmospheric gas regulation (Biospherix, Parish, NY, USA) (Rodriguez et al., 2023). Sensors in the chambers monitored the oxygen concentration. Room air and nitrogen were cycled within the chamber until each defined oxygen set point was reached. The CIH protocol cycled between room air (21% O_2_, 79% N_2_) and hypoxia (6% O_2_, 94% N_2_) 10 times per hour in four phases: (1) room air to hypoxia in 90 s, (2) maintained hypoxia for 120 s, (3) hypoxia to room air in 90 s, and (4) maintained room air for 90 s. This CIH protocol closely imitates the hypoxic events that occur during obstructive events in OSA but does not include the hypercapnia that often also occurs with apnoeas and hypopnoeas. Non‐CIH (room air) animals, inside their cages with normal bedding and unrestricted access to food and water, were placed in the room close to the hypoxia chambers so that animals in both groups experienced the same conditions (sound, luminosity, cage transportation, animal handling and experimental procedures). Animals were returned to the vivarium each day at the end of the CIH protocol and continued this regimen for 26 weeks (timeline: Figure 1).

### 2.4 Morris water maze training

After 26 weeks of exposure to either room air or CIH, each animal underwent an MWM) training protocol to assess both memory and cognitive function. The MWM consisted of a large circular pool (height: 80 cm; diameter 200 cm) that was 80% filled with room temperature (∼20°C) water. The pool was placed in the centre of a 4 × 4 m area with black walls. Attached to the walls were visual cues of varying shapes and colours. The pool was divided into four equally sized quadrants, and a 15.24 cm diameter circular platform was randomly positioned within one of the quadrants, which was defined as the target quadrant.

All MWM training and tests occurred between 10.00 h and 14.00 h, during the light phase of the animal's circadian rhythm. Ten training trials were conducted over a 5‐day period, with two trials occurring each day, for each animal. The platform was elevated 3 cm above the water level to make it visible to the animal. During each trial, animals would swim to the platform after being placed into each of the three non‐target quadrants. Trials began by gently lowering an animal into the water at the edge of a non‐target quadrant, with the animal facing the boundaries of the pool. Animals would then swim and explore the pool to find the platform for up to 60 s. Once the platform was identified, the animals were given 15 s to stand on the platform. The time from the animal entering the water to when it stood on the platform was recorded. If an animal did not reach the platform within 60 s, it was manually guided to the platform and then allowed to stand on it for 15 s. This process was repeated for the remaining two non‐target quadrants to complete the trial. The three time‐to‐platform recordings were averaged (latency ± SD) and used as the latency time for each trial. Differences in latency between groups were assessed by repeated measures ANOVA with subsequent multiple comparisons. No exclusion criteria were set for the acquisition and analysis of these experiments.

### 2.5 Water maze testing

Eight weeks after water maze training, animals were tested using a similar protocol to assess their memory of the water maze. To impede the visibility of the platform to the animals, non‐toxic grey paint (Crayola, Easton, PA, USA) was added to the pool to make the water opaque and the platform was placed 2 cm below the water. A video camera was positioned above the pool to record each test. The time (latency ± SD) the animal took to reach the platform from each of the three non‐target quadrants was recorded and averaged. The position and trajectory of each animal in the pool were extracted from the video recordings using Any‐maze software (Stoelting Co., Wood Dale, IL, USA).

### 2.6 Open field testing

At 46 weeks of age, open field tests (timeline Figure 1) assessed whether anxiety levels were higher in CIH animals compared to non‐CIH animals. Open‐field assessments were performed during the animals’ light phase. Tests were performed using a 1 × 1 m black Plexiglas platform with 40 cm walls. Animals were acclimated to the experimental room for 2 days prior to testing. Each test began by placing an animal in a corner of the platform. A video camera above the platform recorded the animal's unrestricted movement for 10 min. Video recordings were analysed using Any‐maze software to track the movement of the animal within the platform. The time spent by the animal close to the edge of the platform (defined as the area within 15 cm of a wall) and in the centre of the platform (defined as the area greater than 15 cm from a wall) was measured and the ratio of the centre time to the edge time was computed.

### 2.7 Plethysmography

At 46 weeks of age, unrestrained respiratory function (timeline Figure 1) was measured between 10.00 h and 14.00 h, using a whole‐body plethysmography chamber (SCIREQ Scientific Respiratory Equipment Inc., Montréal, QC, Canada). Animals were acclimated by placing them in the chamber (without access to food or water) for 3 days for 3 h each day. On the day of the plethysmography recording, animals were acclimated for 1 h in the chamber prior to a 1‐h recording of respiratory pressure signals. A camera simultaneously recorded a video of the animal during the test. The duration of each breath was measured from the pressure signals using Labchart software (ADInstruments, Colorado Springs CO, USA). Apnoeas were identified as a breath duration twice as long as the mean breath duration over the 1 h of recording. The acquisition software (IOX2 software, EMKA Technologies, Sterling, VA, USA) derived respiratory parameters from the pressure signal, which were averaged across the 1 h period (Table 1).

**TABLE 1:** Calculated respiratory parameters.

Respiratory parameter	CIH	Non‐CIH	*P*
Inspiration time (*T*_i_) (ms)	276.2 ± 29.29	239.6 ± 35.75	0.083
**Expiration time (*T*_e_) (ms)**	**476.5 ± 53.71**	**428.1 ± 42.80**	**0.066**
*T*_i_/*T*_e_	0.5843 ± 0.076	0.56 ± 0.064	0.5013
**Peak inspiratory flow (mL/ms)**	**12.76 ± 3.04**	**15.63 ± 3.17**	**0.0858**
Peak expiratory flow (mL/ms)	9.00 ± 1.92	10.73 ± 2.89	0.1815
Tidal volume (mL)	1.92 ± 0.33	2.07 ± 0.26	0.3444
Expired volume (mL)	1.90 ± 0.33	2.07 ± 0.25	0.2769
Relaxation time (ms)	317.9 ± 50.53	283.5 ± 29.30	0.118
**Minute volume (mL)**	**173.9 ± 41.00**	**217 ± 48.70**	**0.0759**
Frequency (bpm)	99.84 ± 13.77	120.9 ± 31.74	0.1605
End inspiratory pause (ms)	4.366 ± 1.03	4.14 ± 0.75	0.2345
End expiratory pause (ms)	41.15 ± 6.81	37.6 ± 4.09	0.2267

*Note*: Respiratory parameters (mean ± SD) were acquired through whole‐body plethysmography to evaluate the effects of CIH on respiration. No significant differences between groups.

### 2.8 Tissue collection

Prior to terminal assessments, animals were fasted for 16 h to normalize metabolites prior to blood collection. Animals were then anaesthetized with 5% isoflurane inhalation, until the cessation of pedal reflex. Blood was drawn from the inferior vena cava, which was accessed through a longitudinal abdominal incision. Drawn blood was subsequently placed into sodium citrate tubes (Vacutainer, BD Biosciences, San Jose, CA, USA) for the measurement of haematocrit after centrifugation (10,000 *g*; 6 min). Low‐quality samples from poor blood draws were excluded. A thoracotomy was performed to expose the heart, allowing for cardiac perfusion with ice‐cold 1× phosphate‐buffered saline (PBS; Thermo Fisher Scientific, Waltham, MA, USA). Brains were excised; the left and right hemispheres of the hippocampus were isolated, flash‐frozen, and stored at −80°C.

### 2.9 Amyloid beta and tau peptide enzyme‐linked immunosorbent assays

Left hemisphere hippocampus samples were pulverized on dry ice. Using a glass homogenizer, the powder was homogenized in ice‐cold 1× PBS (Thermo Fisher Scientific, 1:9 ratio), supplemented with a protease and phosphatase inhibitor cocktail (Sigma‐Aldrich, St Louis, MO, USA). Samples were sonicated and centrifuged at 5000 *g* for 5 min. The levels of amyloid beta 1–42 and tau peptides were measured using kits from Novus Biologicals (Centennial, CO, USA; tau: NBP2‐81164, amyloid‐beta: NBP2‐69916).

### 2.10 Amyloid beta chemiluminescence measurement

Right hemisphere hippocampus samples were homogenized in 5 M guanidine–HCl solution (Calbiochem ULTROL Grade 369075, MilleporeSigma, Burlington, MA, USA) with a protease and phosphatase inhibitor cocktail (Sigma‐Aldrich) to detect the insoluble hippocampal amyloid beta peptide. The homogenate was shaken for 90 min at 4°C followed by a freeze‐thaw cycle at −20°C. Samples were diluted 1:10, vortexed and centrifuged at 20,000 *g* for 20 min at 4°C. BCA analysis was performed to normalize tissue concentration (Thermo Fisher Scientific). Insoluble amyloid beta was measured using an MSD V‐PLEX Aβ Peptide Panel 1 (4G8) kit (Meso Scale Diagnostics, Rockville, MD, USA).

### 2.11 Statistics

Data were analysed using GraphPad Prism 10.1.0 (GraphPad Software, Boston, MA, USA). Unless otherwise noted, Student's unpaired *t*‐test was used to evaluate differences between groups. The Shapiro–Wilk test was used to test for normality. When appropriate, a Mann–Whitney test was applied. Descriptive statistics are presented as a mean ± standard deviation with a *P*‐value < 0.05 indicating statistical significance between groups.

## 3 RESULTS

### 3.1 Body weight after CIH exposure

CIH and non‐CIH animals were fed a high‐fat diet starting at 12 weeks of age, 4 weeks after ovariectomy. At 46 weeks of age, the mean weight at the time of sacrifice for non‐CIH animals was significantly greater than for CIH animals (non‐CIH: 578.38 ± 81.49 g and CIH: 490 ± 66.90 g, *P* = 0.0379). According to growth charts published by Hilltop Lab Animals, Inc. (Scottdale, PA, USA) these weights, respectively, are 63% and 38% greater than the mean weight of age‐matched female Sprague–Dawley rats fed a diet of standard chow.

### 3.2 Effect of CIH on learning

**FIGURE 2:**
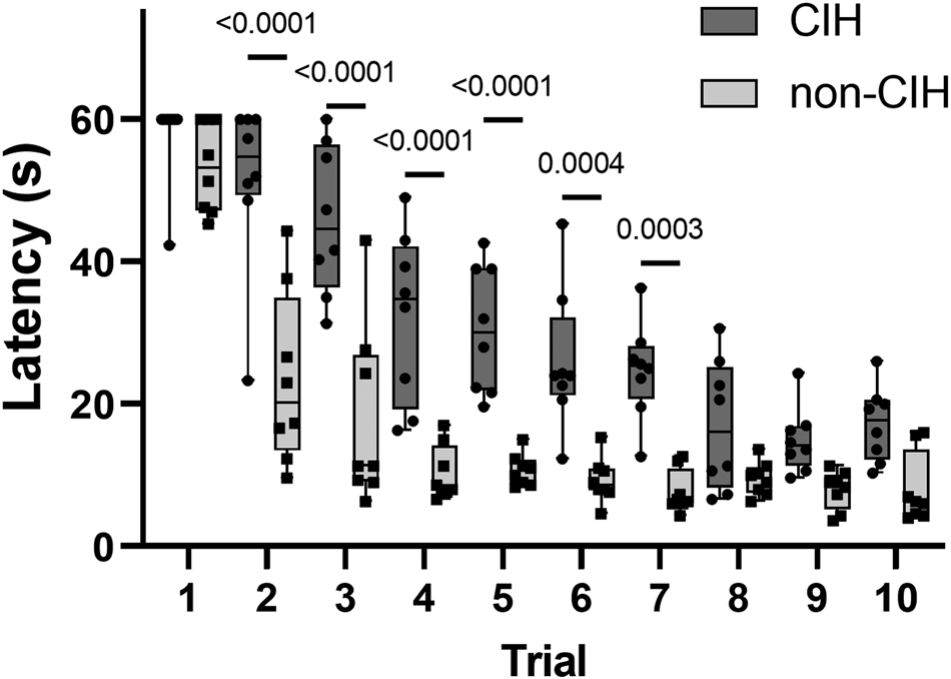
Water maze training. Mean time taken (latency ± SD) by the experimental animals to find the platform across 10 training trials. CIH and non‐CIH animals showed statistically significant differences in latency in Trials 2–7 with two levels of significance (RM ANOVA); *n* = 8 for each group. CIH, chronic intermittent hypoxia.

The effect of chronic intermittent hypoxia on cognition in ovariectomized SD rats was assessed using a spatial memory task during MWM training. A significant difference was observed in the learning trajectories between the groups (Figure 2). Throughout the 10 trials, both CIH and non‐CIH rats displayed reduced latency and distance travelled; however, the mean time for CIH animals to reach the platform was significantly longer for Trials 2–7 (Trials 2–5, *P *< 0.0001 and Trials 6 and 7, *P* = 0.0004), indicating cognitive deficiencies in learning compared to non‐CIH animals.

### 3.3 Effect of CIH on memory

**FIGURE 3:**
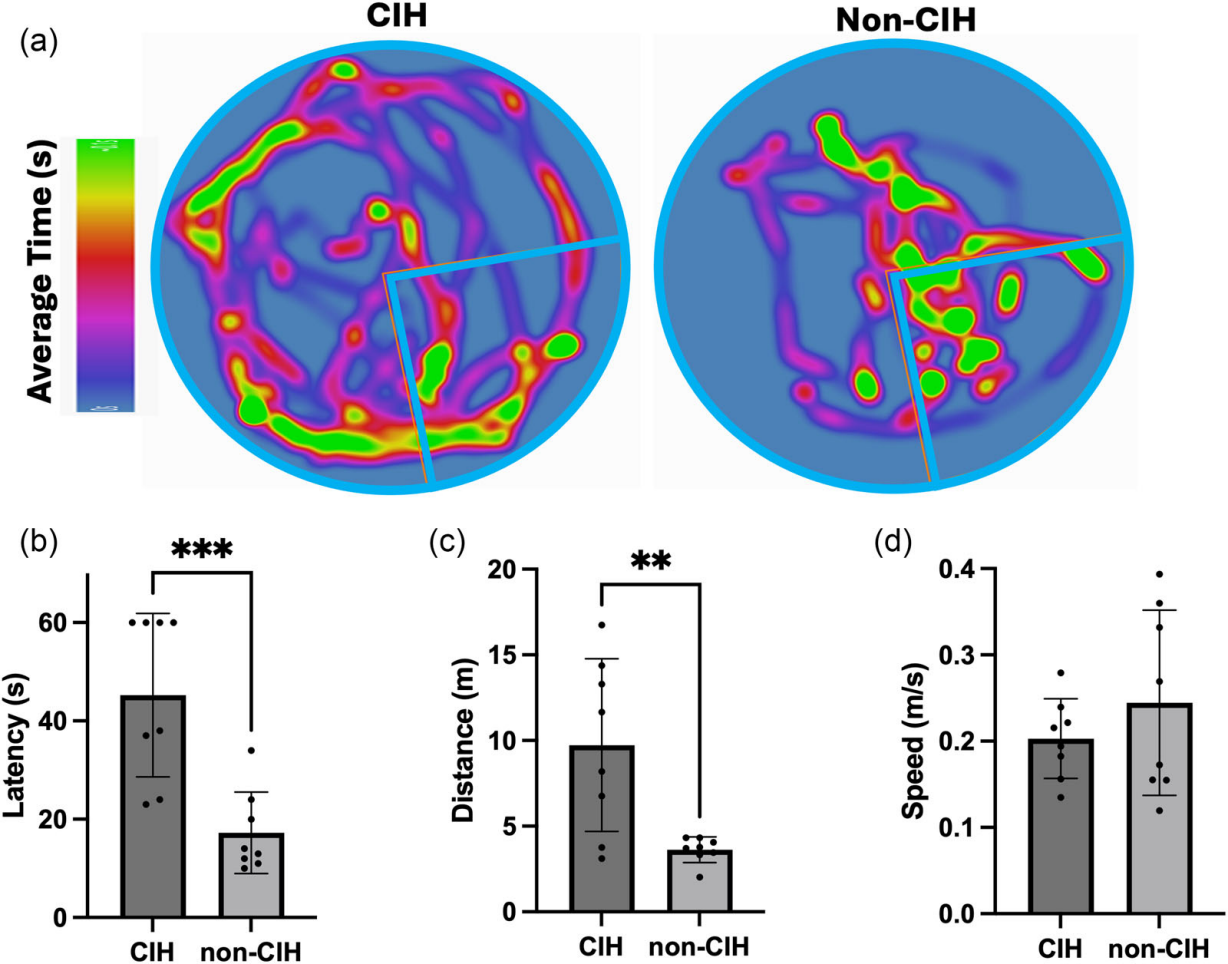
Water maze test. (a) Heat map representation of the time and trajectory of experimental animals while searching for a hidden platform in the MWM test. The quarter of the pool containing the hidden platform target quadrant is marked at the lower right corner of each figure. (b) Mean time taken (latency, ±SD) by the experimental animals to find the hidden platform. Differences in latency between CIH and non‐CIH animals were statistically significant (unpaired *t*‐test, *P* = 0.0008). (c) Mean distance to the platform (±SD) was statistically significant(t‐test, *P* = 0.0044). (d) Mean speed to the platform, calculated by dividing distance by latency; *n* = 8 for each group. CIH, chronic intermittent hypoxia; MWM, Morris water maze.

**FIGURE 4:**
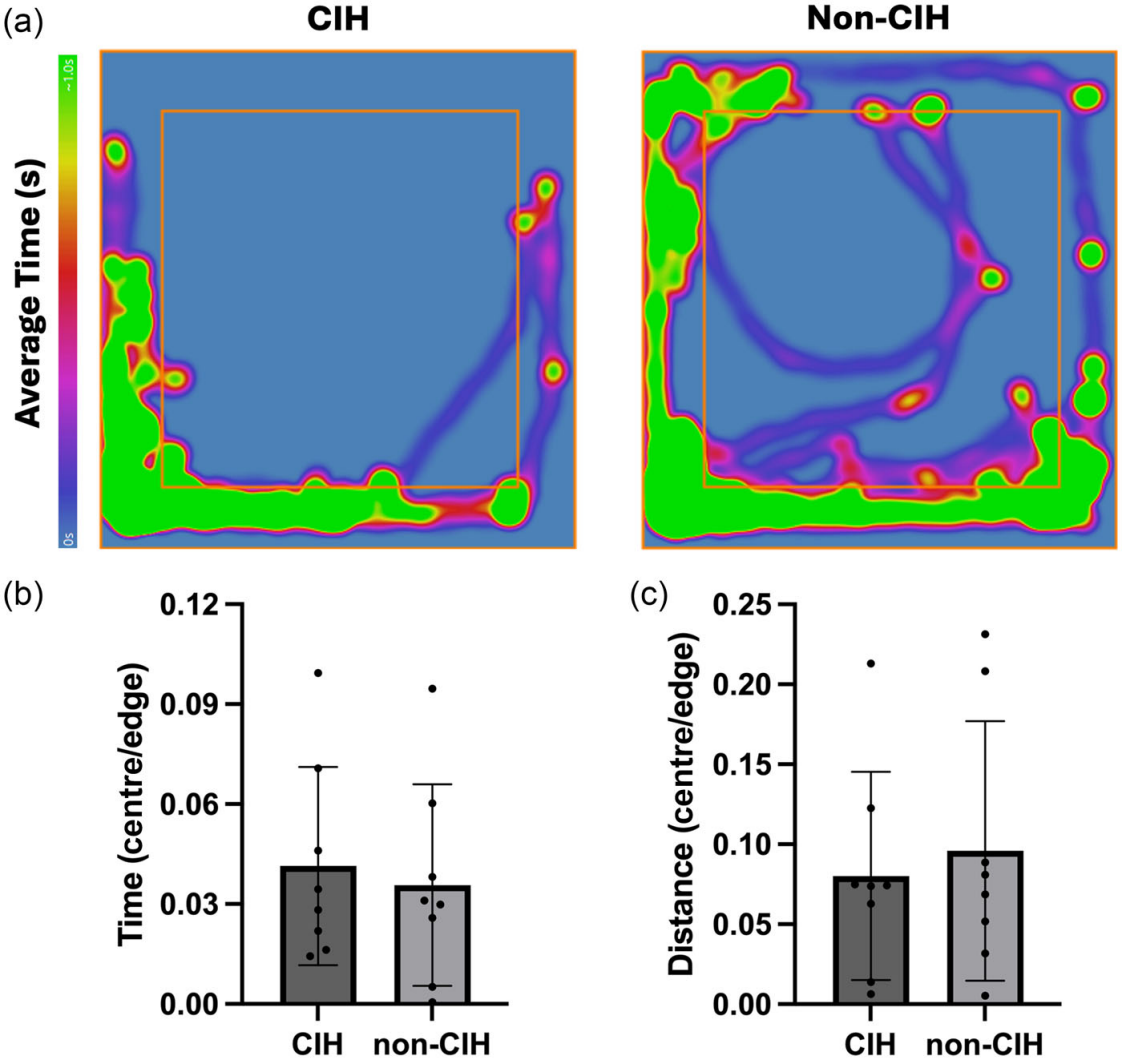
Open field test. (a) Heat map representation of the time and trajectory of experimental animals during the open field test. The zones defined as ‘edge’ and ‘centre’ are delimited by the inner red square. (b, c) Mean proportion of time and distance spent at the edge and centre of the open field experimental arena (±SD); *n* = 8 for each group.

Following the water maze training, animals previously exposed to CIH continued their exposure for 8 h per day. Eight weeks after training, animals were re‐assessed using the MWM to determine their ability to recall the platform location. CIH animals exhibited significantly higher latencies to reach the platform, compared to the non‐CIH animals (Figure 3), suggesting that CIH impaired spatial memory. The open‐field test, a measure of anxiety and exploratory behaviour, did not reveal any differences between groups (Figure 4).

### 3.4 Tau peptide and haematocrit

**FIGURE 5:**
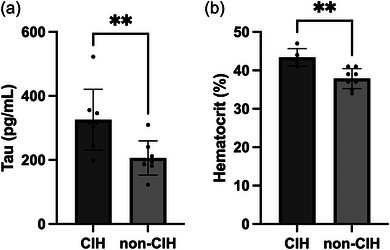
Tau quantification and haematocrit. (a) Protein quantification of the hippocampus revealed that CIH‐exposed animals had higher protein levels of total tau (mean ± SD; CIH: 325.7 ± 95.12 pg/mL, non‐CIH: 206.1 ± 53.48 pg/mL, unpaired *t*‐test, *P* = 0.0078). (b) Blood samples revealed that exposure to CIH increased haematocrit (mean ± SD; CIH: 43.4 ± 2.302%, non‐CIH: 37.88 ± 2.588%, unpaired *t*‐test, *P* = 0.0047); *n* = 8 for each group. CIH, chronic intermittent hypoxia.

Compared to non‐CIH animals, CIH animals exhibited impaired cognition and spatial memory, demonstrated by significantly greater latency to the platform during the water maze training and testing. To determine whether these differences were associated with molecular differences in brain tissue, hippocampus sections were probed for two hallmark proteins of Alzheimer's disease: tau protein and amyloid beta peptide. Enzyme‐linked immunosorbent assay analysis of isolated hippocampal tissue indicated that tau was significantly higher in CIH animals (mean ± SD; CIH: 325.7 ± 95.12 pg/mL, non‐CIH: 206.1 ± 53.48 pg/mL, unpaired *t*‐test, *P* = 0.0078) (Figure 5a). However, amyloid β‐42 was not significantly different between the groups. This was true for both the soluble and insoluble fraction of amyloid beta. These results suggest a correlation between long‐term exposure to daily CIH and higher levels of tau protein, a correlation supported by previous studies (Gao et al., 2013). Blood samples revealed that the haematocrit (%) of CIH animals was significantly higher than non‐CIH animals, demonstrating an adaptive response to CIH resulting in increased erythrocyte production (Figure 5b).

### 3.5 Frequency of apnoeic events

**FIGURE 6:**
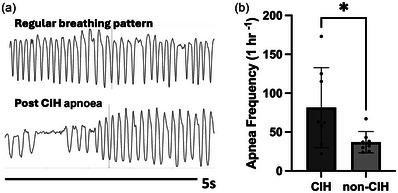
Long‐term CIH contributes to respiratory dysfunction. (a) Graphic representation (obtained from raw plethysmography data) of a single apnoeic event in an animal previously exposed to CIH. (b) Whole body plethysmography revealed that, compared to non‐CIH animals, CIH animals had increased apnoea frequency (mean ± SD; CIH: 81.50 ± 51.35, non‐CIH: 37.00 ± 13.75, unpaired *t*‐test: *P* = 0.0328); *n* = 8 for each group. CIH, chronic intermittent hypoxia.

Increased apnoeic events have been observed in animals exposed to long‐term CIH (Joseph et al., 2020; Souza et al., 2015), and thus we compared respiratory function between CIH and non‐CIH animals using plethysmography. Pressure signals corresponding to a regular breathing pattern and an interval containing an apnoea are plotted in Figure 6a. An apnoeic breath was defined as a breath having a duration of at least twice that of a normal breath. The mean number of apnoeic breaths per hour was measured for each animal and was found to be significantly higher for CIH animals compared to non‐CIH animals (mean ± SD; CIH: 81.50 ± 51.35, non‐CIH: 37.00 ± 13.75, unpaired *t*‐test, *P* = 0.0328 (Figure 6b). Baseline respiratory parameters, with no significant differences between groups, are summarized in Table 1.

## 4 DISCUSSION

Obstructive sleep apnoea (OSA) is a breathing disorder characterized by frequent episodes of partial or complete obstruction of the upper airway during sleep, causing intermittent oxygen desaturations. Compared to men, women remain underdiagnosed and under‐represented in clinical trials related to OSA, which can be attributed to differences in presenting symptoms, variation in polysomnographic findings and sociocultural factors (Geer & Hilbert, 2021). Diagnosis of OSA in postmenopausal women is three‐fold higher compared to premenopausal women, suggesting that sex hormone changes may play a role in the development of OSA (Appiah et al., 2024).

OSA in females is linked to ageing, obesity, menopause, hypertension and cognitive impairments that are associated with AD. To model these clinical risk factors, ovariectomy (OVX) was performed in SD rats to induce menopause and accelerate biological ageing while also feeding the animals a high‐fat diet to increase body mass index. Chronic daily CIH was used to mimic the blood oxygen desaturations that are associated with OSA.

### 4.1 Respiratory changes in ovariectomized females in response to CIH

We did not observe significant changes in baseline respiratory parameters in response to CIH. This is consistent with other whole‐body plethysmography studies in female rats that demonstrate no changes in tidal volume, minute ventilation and breathing frequency after CIH exposure (Skelly et al., 2012; Souza et al., 2015). Interestingly, despite a lack of differences in baseline breathing, we did observe increased apnoea frequency (81.50 ± 51.35 in CIH and 37.00 ± 13.75 in non‐CIH) in CIH‐exposed females, consistent with prior studies in female rats (Souza et al., 2015). This marked increase in apnoeic events in CIH‐exposed animals can be attributed to the cumulative effects of ovariectomy, high‐fat diet and length of CIH exposure. Similar findings by Joseph et al. (2020) demonstrated that OVX females exposed to CIH had increased apnoea and sigh frequency compared to intact females, which correlated with oxidative stress in the brain cortex and brainstem. Likewise, a study in SD males demonstrated that high‐fat diet increased the incidence of apnoeas during non‐REM and REM sleep stages, independent of the development of obesity (Ramadan et al., 2007). Findings from our respiratory data suggest that CIH further augments the incidence of apnoeas in animals fed a high‐fat diet.

### 4.2 Cognitive changes in ovariectomized females in response to CIH

There is a growing body of evidence supporting the relationship between OSA and AD. Our study design highlights the increased risk of cognitive decline that may result from long‐term exposure to CIH. After 26 weeks of exposure to CIH, animals had deficits in spatial learning and subsequent memory impairment. During the training phase, CIH animals displayed significantly longer latencies than non‐CIH to reach the platform, demonstrating deficits in spatial navigation and reference memory. Similar reports in SD males have demonstrated the effects of global hypoxia (30 days of 10% O_2_), where chronic sustained hypoxia exposure led to significant latencies to find a hidden platform (Lei et al., 2022). Importantly, they highlighted the role of chronic hypoxia in upregulating the transcription factor HIF‐1a and how HIF‐1a knockdown restored learning and memory faculty in chronic hypoxia‐exposed rats. Other groups have used MWM to test cognitive performance deficits in response CIH, albeit shorter timeframes of exposure, yielding variable results in latency to reach the platform (Correia et al., 2021; Gao et al., 2013; Goldbart et al., 2003; Gozal et al., 2003). Our data suggests that extended exposure to CIH during sleep accentuated learning deficits and subsequent memory impairment in ovariectomized females fed a high‐fat diet. This view is supported by a recent study in females that observed increased circulating inflammatory marks after 14 days of CIH, but no significant effects on spatial learning and memory (Mabry et al., 2024). Similar to our findings, they also did not observe that CIH influenced anxiety‐like behaviours in the open field test. Furthermore, our preclinical model of cognitive dysfunction in response to long‐term exposure to repeated oxygen desaturations could model untreated sleep apnoea, which affects an estimated 34% of OSA patients who do not comply with the current standard of treatment, CPAP (Gabryelska et al., 2021).

Analysis of hippocampal tissue for hallmarks of Alzheimer's disease revealed an accumulation of total tau in CIH‐exposed animals but not amyloid beta. The CIH‐exposed hippocampi did not show increased amyloid β‐42, despite amyloid β‐42 being a core biomarker of AD. Other studies have examined oestrogen deficiency in ovariectomized APP23 mice and have concluded that brain oestrogen deficiency accelerates amyloid beta plaque formation (Yue et al., 2005). Therefore, because both groups underwent ovariectomy at 8 weeks, they may have similar hormone signalling deficiencies leading to indistinguishable levels of amyloid β‐42 in hippocampi. Clinical data evaluating cerebrospinal fluid of OSA patients reported increased tau protein in the cerebrospinal fluid, which is consistent with our animal model. Surprisingly, they also discovered that OSA patients had lower levels of amyloid β‐42 compared to controls and OSA patients treated with CPAP (Liguori et al., 2017). Thus, work must continue to uncover the underlying mechanisms that govern the relationship between markers of Alzheimer's disease and OSA, particularly in females.

### 4.3 Limitations

Rats were exposed to daytime CIH to align with their sleep cycle; however, we recognize that CIH exposure during the sleeping period can also induce sleep disturbances and sleep fragmentation. To minimize the differences in groups due to sleep fragmentation, we exposed the non‐CIH animals to the same room conditions (sound, luminosity, cage transportation, animal handling and experimental procedures) but without oxygen desaturations. We also recognize that CIH exposure does not mimic all aspects of OSA, including hypercapnia and transthoracic pressure swings (Drager et al., 2011). We did not include males in this study, whose obesity phenotype and associated cognitive function could differ from obese, ovariectomized females. Thus, implementing this protocol in males would be necessary to determine if the results are generalizable to both sexes. Another limitation of our study is that animals were exposed to environmental stressors inherent to behavioural tests (swimming for the water maze test) and fasting prior to tissue collection. While these tasks were essential to assess cognitive function and for measuring metabolic markers, they may have introduced confounding effects that potentially impact the generalizability of the findings.

Our animal model demonstrates a correlation between cognitive dysfunction and markers of Alzheimer's disease; however, the mechanisms of how the cumulative effects of hypoxia exposure, a high‐fat diet, and menopause cause cognitive decline were not studied. Unravelling the interplay of these variables in the development of cognitive dysfunction and identifying therapeutic targets are the goals of future experiments. Finally, when homogenizing hippocampal tissue, we included both the dorsal and ventral portions of the hippocampus, which contain different cognitive centres, including learning memory and mood, respectively; thus, we cannot discern whether protein expression was different between these two regions.

### 4.4 Conclusions

Our results demonstrate a strong relationship between chronic CIH, respiratory dysfunction, and cognitive decline in ovariectomized female rats. Compared to rats not exposed to CIH, CIH‐exposed rats had significant deficits in spatial learning and memory, increased expression of hippocampal total tau protein and increased frequency of apnoeas. The neurological mechanisms responsible for CIH‐mediated cognitive decline and increased hippocampal total tau in ovariectomized female rats remain to be identified. With the increasing prevalence of OSA and AD, it is important to understand how ageing‐associated diseases are linked, to develop new therapies that reduce the cognitive burden on patients while simultaneously addressing both conditions. Overall, our results provide new preclinical evidence of the association between OSA, cognitive decline and AD pathology in females, emphasizing the importance of sex‐specific research in understanding and addressing these pathophysiological interconnections.

## AUTHOR CONTRIBUTIONS

Conceptualized experiments: Emily C. Cheung, Joan B. Escobar, David Mendelowitz, Matthew W. Kay, Kathryn Schunke, and Vivek Jain. Performed daily CIH exposures and plethysmography: Emily C. Cheung, Joan B. Escobar, Jeannette Rodriguez, Grey Harral, John T. Ketzenberger, Aman Gill, and Makeda Melkie. Performed end of study experiments: Emily C. Cheung, Grant Kowalik, and Bridget R. Alber. Performed protein quantification and data analysis: Emily C. Cheung, Joan B. Escobar, and Caitlin Ribeiro. Guided data interpretation: David Mendelowitz, Matthew W. Kay, Vsevolod Y Polotsky, John Bethea, and Kathryn Schunke. Drafted the manuscript: Emily C. Cheung, Joan B. Escobar, Kathryn Schunke, Matthew W. Kay, and David Mendelowitz. All authors have read and approved the final version of this manuscript and agree to be accountable for all aspects of the work in ensuring that questions related to the accuracy or integrity of any part of the work are appropriately investigated and resolved. All persons designated as authors qualify for authorship, and all those who qualify for authorship are listed.

## GAI TOOLS

Chat‐GPT4 was used to generate *only* the abstract of this manuscript. The entire contents of the manuscript were inserted into Chat‐GPT4 and it was prompted to ‘write an abstract of less than 250 words.’ Subsequent editing of the generated content by the authors resulted in the final version.

## CONFLICT OF INTEREST

None declared.

## Data Availability

Upon a reasonable request, analysed datasets are available from the corresponding author.
